# Efficacy and Safety of Safflower Yellow in Early Diabetic Nephropathy: A Meta-Analysis

**DOI:** 10.1155/2019/8065376

**Published:** 2019-02-14

**Authors:** Xiuze Jin, Liuyan Shi, Feng Chang, Yun Lu

**Affiliations:** School of International Pharmaceutical Business, China Pharmaceutical University, Nanjing, Jiangsu, China

## Abstract

**Background:**

Diabetic nephropathy (DN) is a major cause of end-stage renal disease. In order to palliate renal function impairment and reduce kidney related mortality, it is crucial to treating DN patients at the early stage. This study aims to assess the efficacy and safety of conventional therapy combined with safflower yellow versus conventional therapy alone in early DN patients.

**Methods:**

A meta-analysis of randomized controlled trials that compared safflower yellow plus conventional therapy with conventional therapy alone in early DN patients was conducted. Papers were searched using the electronic databases and reference lists. Two reviewers working independently extracted relevant data and carried out risk-of-bias assessments. Statistical analysis was undertaken in Review Manager 5.3.

**Results:**

Fourteen trials (1,072 patients) were included in the meta-analysis. Conventional therapy combined with safflower yellow was associated with a higher effective rate (RD, 0.24; 95% CI, 0.17 to 0.30) and a greater decline in urinary albumin excretion rates (SMD, -1.34; 95% CI, -1.77 to -0.92), fasting blood glucose (MD, -0.57; 95% CI, -0.98 to -0.16), serum creatinine (MD, -12.36; 95% CI, -14.66 to -10.06), and blood urea nitrogen (SMD, -0.93; 95% CI, -1.13 to -0.73) in the subgroup with a follow-up time > 15 days. The incidence of adverse events did not differ significantly between these two regimens (RD, -0.01; 95% CI, -0.03 to 0.01). Findings were similar in the subgroup with a follow-up time < 15 days.

**Conclusions:**

Conventional therapy combined with safflower yellow had a more beneficial effect than conventional therapy alone in early DN patients. There were significant differences in effective rate, urinary albumin excretion rates, fasting blood glucose, serum creatinine, and blood urea nitrogen between the two regimens and no significant difference in adverse events. More randomized controlled research using standardized protocols would be needed in the future to compare these two regimens.

## 1. Introduction

Diabetic nephropathy (DN), one of the most common microvascular complications in Diabetes Mellitus (DM), has become a major cause of end-stage renal disease (ESRD) [1–4]. The symptoms of DN include decreased glomerular filtration rate, small amounts of albuminuria, elevated arterial blood pressure, proteinuria and fluid retention, and renal failure. It is crucial to treating DN patients at the early stage to palliate renal function impairment and reduce kidney related mortality. Although interventions, such as diet control, glycemic control, blood pressure control, and inhibition of the renin-angiotensin-aldosterone system, have been shown to postpone the development of disease, mortality of DN remains high and has increased significantly from 2005 (299.4 thousand) to 2015 (417.8 thousand) [5].

Previous studies [6–12] have demonstrated that the inflammation pathways play important roles in the progression of diabetic nephropathy. Anti-inflammatory drugs may delay the progression of DN from the level of cytokines [13]. The Traditional Chinese Medicine (TCM), safflower yellow, is associated with promoting blood circulation, antioxidation, and anti-inflammatory effect, and it has been used to protect renal function in daily clinical practice [14]. This study aims to assess the efficacy and safety of conventional therapy combined with safflower yellow compared with conventional therapy alone in early DN patients.

## 2. Materials and Methods

### 2.1. Data Sources and Search Strategy

We searched Pubmed, Embase, Cochrane Library, China National Knowledge Infrastructure (CNKI), the Chinese Biomedical Literature (CBM), and Wanfang from Jan. 1, 2000 to July 18, 2017. The keywords included in the search strategy were: safflower yellow, early diabetic nephropathy, and diabetic kidney disease. The references of included studies were traced to dig out more relevant studies. We also browsed ClinicalTrial.gov to collect trial results that have not been reported elsewhere.

### 2.2. Inclusion and Exclusion Criteria

RCTs, containing a control group and an intervention group, which fulfill the following criteria were eligible for inclusion:

1. Human studies on adult (≥18 years of age) male or female participants with early diabetic nephropathy.
2. Conventional therapy, including diabetes education, diet, exercise, snfglycemic and blood pressure control, was applied in the control group. Conventional therapy plus safflower yellow was applied in the experimental group.
3. Clinical outcomes (effective rate, urinary albumin excretion rates, fasting blood glucose, serum creatinine, and blood urea nitrogen) were reported.
4. Accessible full-text articles.
5. Languages in Chinese or English.

Studies were excluded if they were

1. not RCTs;
2. with too long follow-up duration (e.g., 6 months);
3. not intravenous infusion administration;
4. duplicate publication.

### 2.3. Data Extraction and Risk-of-Bias Assessment

For each eligible trial, we collected the following information: first author, year of publication, follow-up time, intervention, sample size, patients' baseline characteristics, and key efficacy and safety outcomes. Our primary efficacy outcome was the effective rate, which was the proportion of participants that became markedly improved or improved. “Markedly improved” means that the symptoms of hypertension, proteinuria, and edema disappeared or improved significantly, for example, urinary albumin excretion rates decreased by 1/2 or 40% and fasting blood glucose decreased by 1/3; renal function indexes were all in the normal range at the same time. “Improved” means that all the indexes did not decline as obviously as those mentioned above. Secondary efficacy outcomes included urinary albumin excretion rates (UAER), fasting blood glucose (FBG), serum creatinine (Scr), and blood urea nitrogen (BUN). Safety outcome referred to the incidence of adverse events (nausea, headache, anaphylactic shock, fever, rash, arrhythmia, etc.).

Two authors independently assessed the quality of studies by using the Cochrane risk-of-bias tool [15]. The following items were assessed: (1) selection bias: random sequence generation, allocation concealment; (2) performance bias: blinding of participants and personnel; (3) detection bias: blinding of outcome assessment; (4) attrition bias: incomplete outcome data; (5) reporting bias: selective reporting; (6) other biases.

### 2.4. Statistical Analysis

Meta-analysis was conducted when at least three studies reported relevant outcomes. For dichotomous outcomes, the risk difference (RD) with 95% confidence interval (CI) was calculated. For continuous outcomes, the mean difference (MD) or the standardized mean difference (SMD) with 95% CI were calculated. Data were pooled using the fixed effects model, but the random effects model was also considered to ensure the robustness of the model. The I^2^ statistic was used to quantify heterogeneity, with I^2^ values > 50% representing high heterogeneity. The significance level was set at p < 0.05. Subgroup analysis was conducted for different follow-up time. Statistical analysis was undertaken in Review Manager 5.3 (Cochrane Collaboration, Copenhagen, Denmark).

## 3. Results

### 3.1. Literature Search Results and Study Characteristics

**Figure 1:**
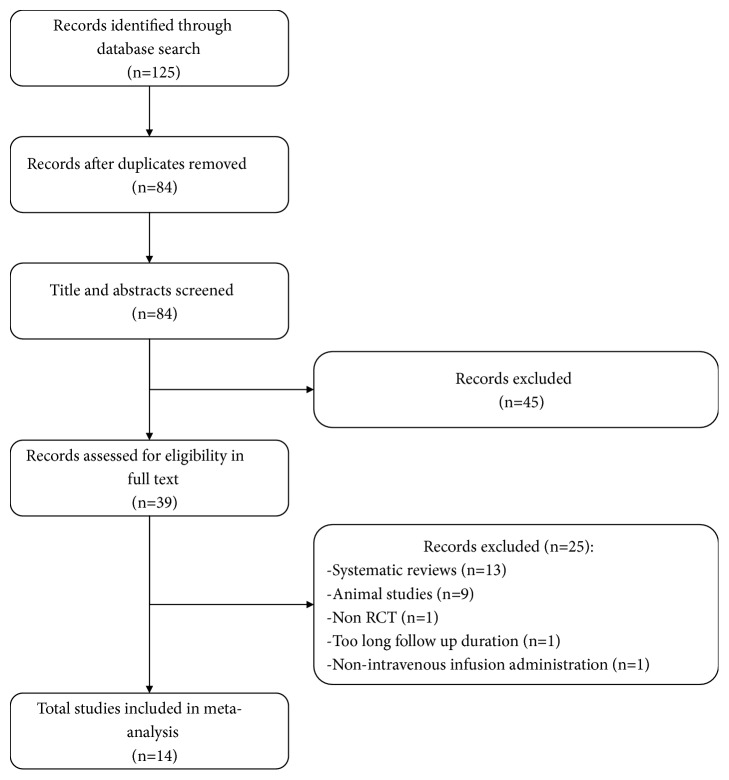
Flow chart of the study selection.

As shown in Figure 1, we identified a total of 125 studies from the initial search, of which 41 studies were removed for duplication. After title and abstract screening, full-texts of the remaining 39 articles were retrieved for detailed review. Finally, 14 studies were included in the present meta-analysis.

Characteristics of the included studies were summarized in Table 1. These 14 studies involved a total of 1,072 DN patients, of whom 538 were treated with conventional therapy, and 534 were treated with conventional therapy plus safflower yellow. The mean age of each study's participants ranged from 46.9 to 65.0 years. The follow-up time varied from 14 days to 12 weeks.

### 3.2. Risk-of-Bias Assessment

**Figure 2:**
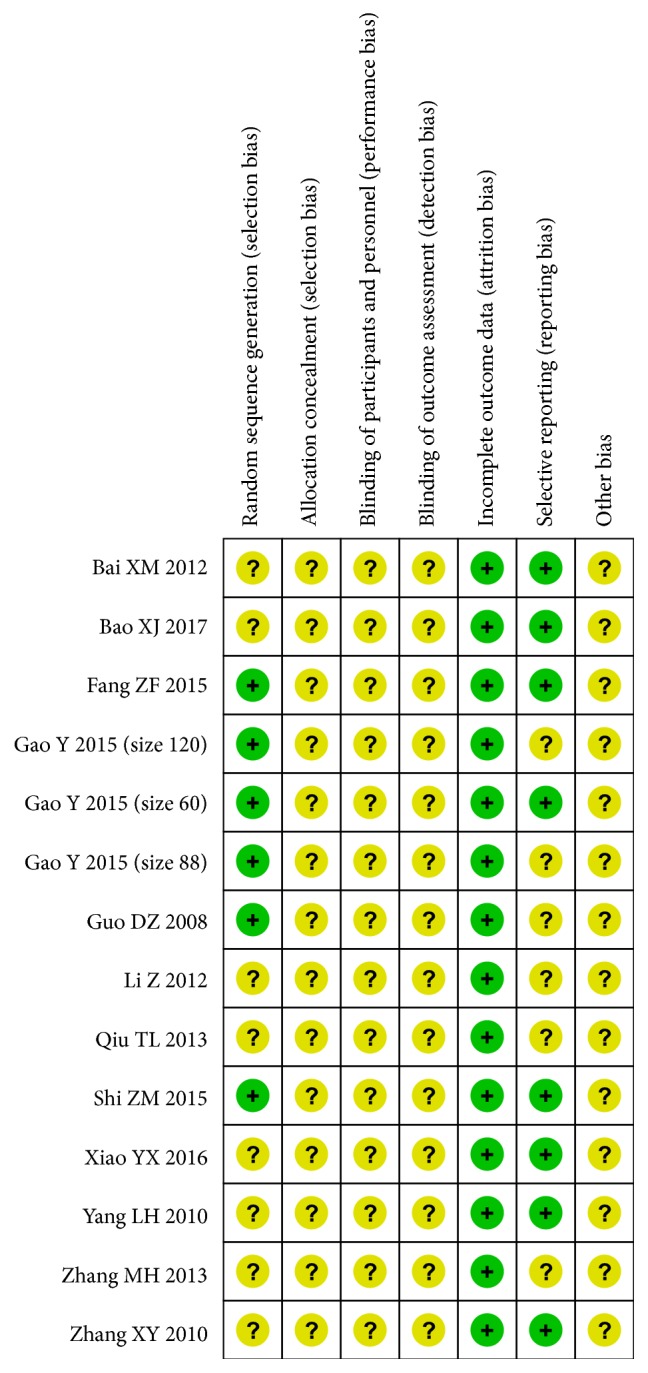
Risk-of-bias summary: authors' judgments about each risk-of-bias item for each included study.

The results of the risk-of-bias assessment were provided in Figure 2. All of the fourteen selected papers were randomized controlled trials. Only six trials [16–21] described the methods of randomization, such as the envelope method and the random figure table. None of the selected trials illustrated the allocation concealment and blinding. No subjects withdrew from the trial. Six trials [16–18, 22–24] did not report adverse events. In addition, there was insufficient information to identify whether there were other potential biases in the selected papers.

### 3.3. Meta-Analysis Result

#### 3.3.1. Effective Rate of Safflower Yellow

**Figure 3:**
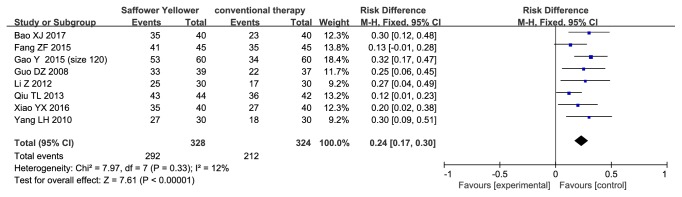
Forest plot displaying the effect of safflower yellow on effective rate.

Eight studies that reported the effective rate were analyzed under a fixed mode (n=652 subjects). The meta-analysis showed a significantly higher effective rate in safflower yellow group compared with that in control group (RD, 0.24; 95% CI, 0.17 to 0.30; p<0.00001; Figure 3). There was no evidence of heterogeneity between these studies (p=0.33; I^2^=12%).

#### 3.3.2. UAER of Safflower Yellow

**Figure 4:**
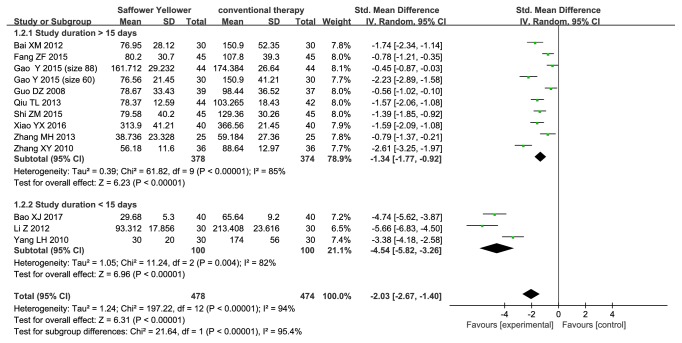
Forest plot displaying the effect of safflower yellow on UAER.

Thirteen trials (n=952 subjects) evaluated UAER (Figure 4). Pooled analysis demonstrated that UAER did decrease significantly in safflower yellow group compared with that in control group (SMD, -1.34; 95% CI, -1.77 to -0.92; p<0.00001 in the subgroup with a follow-up time > 15 days, and -4.54; 95% CI, -5.82 to -3.26; p<0.00001 in the subgroup with a follow-up time < 15 days). However, significant heterogeneity between studies was noted in each subgroup (I^2^=85% in the subgroup with a follow-up time > 15 days, and I^2^=82% in another subgroup).

#### 3.3.3. FBG of Safflower Yellow

**Figure 5:**
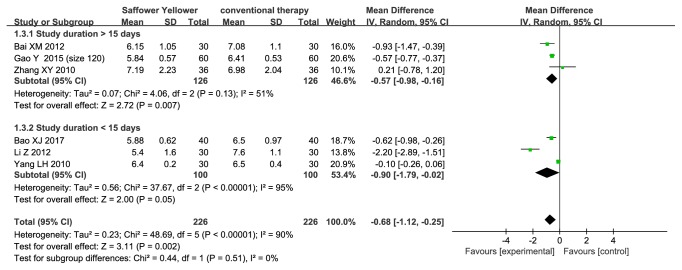
Forest plot displaying the effect of safflower yellow on FBG.

Six trials (n=452 subjects) measured FBG (Figure 5). Compared with control group, safflower yellow group was associated with a significant FBG reduction (MD, -0.57; 95% CI, -0.98 to -0.16; p=0.007 in the subgroup with a follow-up time > 15 days, and -0.90; 95% CI, -1.79 to -0.02; p=0.05 in another subgroup). Heterogeneity among the included studies was significant in the subgroup with a follow-up time < 15 days (p<0.00001, I^2^=95%), while it was not significant in another subgroup (p=0.13, I^2^=51%).

#### 3.3.4. Scr of Safflower Yellow

**Figure 6:**
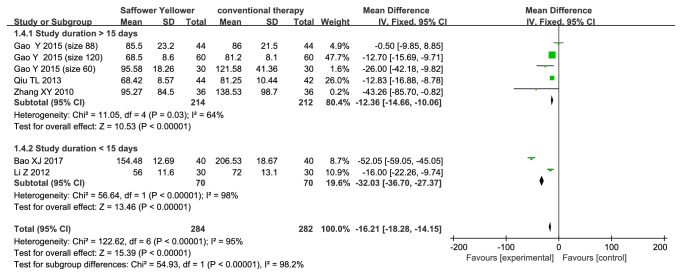
Forest plot displaying the effect of safflower yellow on Scr.

As shown in Figure 6, the effect of safflower yellow on Scr was assessed in seven trials (n=566 subjects). Statistically significant Scr reduction was shown in safflower yellow group (MD, -12.36; 95% CI, -14.66 to -10.06; p<0.00001 in the subgroup with a follow-up time > 15 days, and -32.03; 95% CI, -36.70 to -27.37; p<0.00001 in another subgroup). Significant heterogeneity between studies was noted in each subgroup (I^2^=64% and I^2^=98%, respectively).

#### 3.3.5. BUN of Safflower Yellow

**Figure 7:**
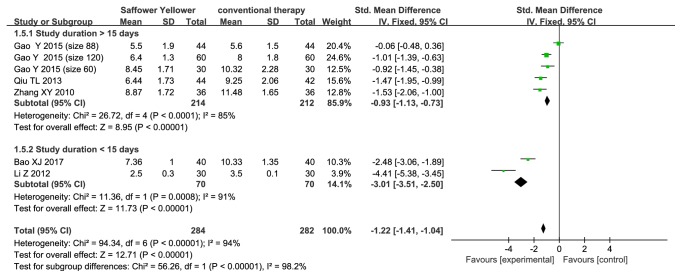
Forest plot displaying the effect of safflower yellow on BUN.

Seven trials (n=566 subjects) examined BUN (Figure 7). We found that BUN was lower in safflower yellow group compared with that in control group, with a pooled SMD of -0.93 (95% CI, -1.13 to -0.73; p<0.00001) in the subgroup with a follow-up time > 15 days, and -3.01 (95% CI, -3.51 to -2.50; p<0.0001) in the subgroup with a follow-up time < 15 days, respectively. Significant heterogeneity between studies was noted in each subgroup (I^2^=85% and I^2^=91%, respectively).

#### 3.3.6. Adverse Events

**Figure 8:**
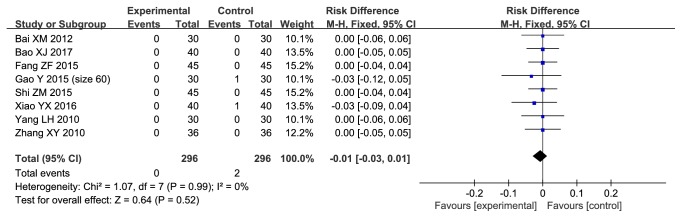
Forest plot displaying the effect of safflower yellow on the adverse event.

Eight included studies (n=592 subjects) reported adverse events. The incidence of adverse events did not differ between safflower yellow group and control group (RD, -0.01; 95% CI, -0.03 to 0.01; p=0.52; Figure 8). There was no evidence of heterogeneity between these studies (p=0.99; I^2^=0%).

## 4. Discussion

In this meta-analysis, conventional therapy combined with safflower yellow not only significantly reduced UAER, FBG, Scr, and BUN, but also was associated with a higher effective rate, compared with conventional therapy alone. For safety outcome, there was no statistical difference between these two regimens. Similar evidence has been produced in previous studies. Yang W J [25] conducted a meta-analysis (n=1,048 subjects) to assess the effect of safflower yellow on UAER and Scr in the elderly who suffered early diabetic nephropathy. The results demonstrated that safflower yellow significantly decreased UAER and Scr level. However, remarkable heterogeneity between selected studies was noted. Cui G N [26] carried out a pooled analysis to evaluate the effect of safflower yellow in a variety of medical conditions, including stable angina, unstable angina, coronary heart disease angina, brain infarction, and diabetic nephropathy (n=268 subjects for DN). The result indicated that safflower yellow reached a higher effective rate. Because of the significant heterogeneity, the author pointed out that meta-analysis could not be adopted to evaluate the efficacy outcome on UAER and FBG.

Our meta-analysis comprehensively estimated more clinical outcomes and included a larger sample size (n=1,072 subjects) than papers published before. Subgroup analysis was carried out to identify the influence of different follow-up time. Nevertheless, heterogeneity between included trials was still conspicuous, in line with previous studies.

There are several limitations of this meta-analysis. Firstly, all of the selected studies were published in Chinese, which might cause publication bias. Secondly, selected trials were all small-scale. Thirdly, the diagnostic criteria of “early diabetic nephropathy” were not entirely consistent: nine trials [17–24, 27] adopted WHO recommended diabetes diagnostic criteria and Mogensen early diabetic nephropathy staging criteria; one trial [16] employed diabetes diagnostic criteria developed by the American Diabetes Association (ADA) in 2010 and diabetic nephropathy diagnosis developed by the Chinese Academy of TCM nephropathy branch in 2008; one trial [28] used early diabetic nephropathy diagnostic criteria from the eighth edition of Internal Medicine in China in 2013; three trials [29–31] did not state in detail which diagnostic criteria were used. This might be an important factor for heterogeneity. Fourthly, the intervention of the control group in some trials was not uniform. There were some differences in the regimen claimed as “conventional therapy”. For example, the antihypertensive drugs differed among some trials [16–19, 21, 22, 28, 29]. Moreover, there was a potential bias in studies [20, 23, 24, 27, 30, 31] that did not specify the drugs and dosage used in conventional therapies. However, subgroup analysis for different “conventional therapies” could not be applied because of insufficient information disclosure. Finally, concerning the results of the quality assessment, there were obvious shortcomings in the study design of included papers. Therefore, more rigorous randomized controlled trials would be needed in the future to confirm our findings.

## 5. Conclusions

In summary, our meta-analysis demonstrated that conventional therapy combined with safflower yellow had a more beneficial effect than conventional therapy alone in early DN patients. The differences between the two regimens were statistically significant on effective rate, UAER, FBG, Scr, and BUN, except for adverse events. However, the quality of the included studies was low. Therefore, more randomized controlled trials using standardized protocols would be required in the future to enhance our understandings of these two regimens.

## Figures and Tables

**Table 1:** Characteristics of included studies (E: experimental group; C: control group).

Num	Authors, publication year [reference]	Number of participants (% female)	Mean age (years)	Follow-up time (days)	Intervention		Outcomes
					Experimental group	Control group	
1	Li Z et al. 2012 [22]	E: 30(28.33) C: 30(28.33)	E: 61.3 C: 61.3	15	Conventional treatment, Losartan potassium 50 mg, safflower yellow 100 mg	Conventional treatment^*∗*^, Losartan potassium 50 mg	①②③④⑤

2	Yang L H et al. 2010 [27]	E: 30(46.67) C: 30(40)	E: 58.3 C: 58.6	14	Conventional treatment, safflower yellow 100 mg	Conventional treatment	①②③⑥

3	Qiu T L et al. 2013 [23]	E: 42(42.86) C: 44(43.18)	E: 51.6 ± 6.6 C: 52.7 ± 7.1	30	Conventional treatment, safflower yellow 100 mg	Conventional treatment	①②④⑤

4	Gao Y et al. 2015 [16]	E: 60(45) C: 60(41.67)	E: 64.6 ± 5.7 C: 65.0 ± 5.8	28	Conventional treatment, Benazepril 10mg, safflower yellow 100 mg	Conventional treatment, Benazepril 10mg	①③④⑤

5	Bao X J. 2017 [28]	E: 40(45) C: 40(42.5)	E: 50.45 ± 7.12 C: 49.74 ± 7.03	14	Conventional treatment, Metformin 2g, safflower yellow 100 mg	Conventional treatment, Metformin 2g	①②③④⑤⑥

6	Zhang X Y. 2010 [29]	E: 36(47.22) C: 36(44.44)	E: 62 C: 64	28	Conventional treatment, Irbesartan 150mg, safflower yellow 100 mg	Conventional treatment, Irbesartan 150mg	②③④⑤⑥

7	Gao Y et al. 2015 [17]	E: 44(42) C: 44(42)	E: 51.13 ± 7.42 C: 51.13 ± 7.42	28	Conventional treatment, Telmisartan 80mg, safflower yellow 100 mg	Conventional treatment, Telmisartan 80mg	②④⑤

8	Xiao Y X. 2016 [30]	E: 40(47.5) C: 40(42.5)	E: 58.9 ± 13.2 C: 59.5 ± 12.4	28	Conventional treatment, safflower yellow 100 ml	Conventional treatment	①②⑥

9	Guo D Z. 2008 [18]	E: 37(48.65) C: 39(48.72)	E: 46.9 ± 13.2 C: 47.3 ± 12.6	35	Conventional treatment, Benazepril 10mg, safflower yellow 150mg	Conventional treatment, Benazepril 10mg	①②

10	Zhang M H. 2013 [24]	E: 25(48) C: 25(48)	E: 56 ± 8.6 C: 56 ± 8.6	28	Conventional treatment, safflower yellow 150mg	Conventional treatment	②

11	Fang Z F. 2015 [19]	E: 45(46.67) C: 45(51.11)	E: 56.0 ± 7.9 C: 55.8 ± 7.6	8 weeks	Conventional treatment, Valsartan 80mg, safflower yellow 100 ml	Conventional treatment, Valsartan 80mg	①②⑥

12	Bai X M. 2012 [31]	E: 30(46.67) C: 30(46.67)	E: 56 ± 7.9 C: 55.8 ± 7.6	30	Conventional treatment, safflower yellow 200mg	Conventional treatment	②③⑥

13	Gao Y et al. 2015 [20]	E: 30(40) C: 30(40)	E: 59.5 ± 12.4 C: 59.5 ± 12.4	30	Conventional treatment, safflower yellow 100mg	Conventional treatment	②④⑤⑥

14	Shi Z M 2015 [21]	E: 45 (48.89) C: 45(48.89)	51.5 51.5	12 weeks	Conventional treatment, Valsartan 80mg/d, safflower yellow 150mg	Conventional treatment, Valsartan 80mg/d	②⑥

^*∗*^Conventional treatment includes diabetes education, diet, exercise, and glycemic and blood pressure control.

①Effective rate, % .

②UAER.

③FBG.

④Scr.

⑤BUN.

⑥Adverse event.
